# Global burden of atrial fibrillation attributable to high body mass index from 1990 to 2021: findings from the Global Burden of Disease Study 2021

**DOI:** 10.1186/s12872-024-04202-5

**Published:** 2024-10-08

**Authors:** Xiangmeng Kong, Mingliang Wang, Yumei Jiang

**Affiliations:** 1 Department of Rehabilitation, Tongji Hospital Affiliated to Tongji University, Tongji University School of Medicine, Shanghai, 200065 China; 2 Department of Cardiology, Putuo People’s Hospital, Tongji University School of Medicine, Shanghai, 200092 China; 3 Department of Cardiology, Shanghai Ninth People’s Hospital, Shanghai Jiao Tong University School of Medicine, Shanghai, 200011 China

**Keywords:** Atrial fibrillation, High body mass index, Global Burden of Disease, Disability-adjusted life years, Mortality

## Abstract

**Objectives:**

To assess the global burden of atrial fibrillation (AF) attributable to high body mass index (BMI) from 1990 to 2021 and analyze its spatiotemporal distribution characteristics.

**Study design:**

An observational study based on GBD 2021 data.

**Methods:**

Data on AF burden due to high BMI were obtained from the Global Burden of Disease Study (GBD) 2021. Estimated annual percentage change (EAPC) was calculated to evaluate temporal trends in age-standardized rates of deaths and disability-adjusted life years (DALYs) over 30 years.

**Results:**

In 2021, high BMI-related AF caused 27,000 deaths and 725,000 DALYs globally, a 376% increase since 1990. Females and the elderly (aged 70+) bore a higher burden. Upper-middle-income regions surpassed high-income regions in AF burden. Australasia had the highest age-standardized rates, while High-income Asia Pacific and South Asia had the lowest. South Asia showed rapid growth in age-standardized death rates.

**Conclusion:**

The global burden of high BMI-related AF varies across regions and time, threatening global health, especially for females and the elderly. Targeted strategies are needed to reduce AF and obesity.

**Supplementary Information:**

The online version contains supplementary material available at 10.1186/s12872-024-04202-5.

## Introduction

Atrial fibrillation (AF), the most prevalent cardiac arrhythmia, disproportionately affects elderly individuals (> 70 years) and those with lifestyle-related comorbidities, including hypertension, diabetes, and obesity [1]. The presence of AF is strongly correlated with heightened morbidity and mortality rates stemming from stroke, heart failure, and dementia [2, 3].

BMI is a commonly used indicator for assessing overweight and obesity, caused by excess body fat is considered risk factors for many health problems, including cardiovascular disease, diabetes, certain cancers, and joint diseases. Over the past few decades, the global prevalence of obesity has increased. In 2013, some countries, such as Oceania, North Africa, and the Middle East, had obesity prevalence rates exceeding 50% of the adult population [4].

High BMI can promote the development and progression of AF through various mechanisms, such as inducing left atrial structural and electrophysiological remodeling, exacerbating myocardial fibrosis, and triggering systemic inflammation [5, 6]. Observational studies have shown that obesity independently increases the risk of newly diagnosed AF [7]. Maintaining sinus rhythm is significantly more challenging in obese individuals, with a 13% increased risk of AF recurrence following radiofrequency ablation for every 5-unit increase in BMI [8–11]. Furthermore, obesity is a significant risk factor for obstructive sleep apnea (OSA), which is independently associated with atrial fibrillation. Research has shown that both obesity and OSA-related nocturnal oxygen desaturation are independent risk factors for incident AF, particularly in individuals under 65, highlighting the complex interplay between these conditions [12, 13]. However, the epidemiological characteristics of the AF burden caused by high BMI have not been investigated globally, particularly in terms of assessing temporal trends, regional differences, and population impact. Therefore, our study aims to summarize the global, regional, and national burden of high BMI in 2021, considering age and sex differences in the disease burden based on deaths and disability-adjusted life years (DALYs), and to estimate the corresponding spatial distribution and temporal trends from 1990 to 2021.

## Method

### Data source

The 2021 GBD study offers an in-depth assessment of health detriments associated with 369 diseases, injuries, and impairments, as well as 88 risk factors, encompassing 204 nations and territories, utilizing the most recent epidemiological data and enhanced standardized methodologies [1]. The original data for this study were sourced from the Global Health Data Exchange (GHDx) online query tool (https://ghdx.healthdata.org/), provided by the Institute for Health Metrics and Evaluation (IHME). The GBD study uses deidentified data, and a waiver of informed consent was re-viewed and approved by the University of Washington Institutional Review Board. All data aggregation and analysis followed the Guidelines for Accurate and Transparent Health Estimates Reporting (GATHER) [14].

### Definition

AF is a common supraventricular arrhythmia characterized by rapid and irregular atrial activation, leading to ineffective atrial contraction. According to the 2020 European Society of Cardiology (ESC) Guidelines, AF is defined by the following electrocardiographic (ECG) features: (1) Absolutely irregular RR intervals, (2) No distinct P waves on the ECG, and (3) An atrial cycle length (when visible) that is usually variable and < 200 ms (> 300 bpm). Clinically, AF is diagnosed by a 12-lead ECG or a single-lead ECG strip showing heart rhythm with these features for at least 30 s. In the GBD 2021 study, high BMI was defined as BMI ≥ 25 kg/m² [15]. ASR are weighted average age-specific rates calculated using the age distribution of a standard population as weights and can serve as a summary measure for comparing rate levels between different populations. DALYs are the sum of YLLs and YLDs for each cause, location, year, sex, and age group [2]. SDI is a composite indicator that measures the socioeconomic development level of each region.

### Statistical analysis

We extracted the number of deaths, DALYs, and corresponding ASRs (per 100,000 persons) related to AF due to high BMI from GBD 2021, including ASDR, age-standardized DALYs rates (ASRDALYs) and their corresponding EAPC, stratified by geographical location, sex, year, and age group (30–94 years at 5-year intervals, and ≥ 95 years). To assess the uncertainty due to the lack of location-, time period-, and heterogeneity-specific data, we calculated the 95% UI for each metric based on the 2.5th and 97.5th percentiles of 1,000 sampled level estimates [16].

We fitted a linear regression model to the natural logarithm of the annual ASR against calendar year (x): ln(ASR) = α + βx + ε. The EAPC and its corresponding 95% confidence interval (CI) were calculated using the following formula: EAPC = 100 × (exp(β) − 1).When the lower limit of the 95% CI of the EAPC was greater than 0, the ASR was considered to have an increasing trend; when the upper limit of the 95% CI of the EAPC was less than 0, the ASR was considered to have a decreasing trend; when the 95% CI contained 0, the ASR trend was considered stable [4].

All procedures for analysis and graphic representation were performed utilizing the World Health Organization’s Health Equity Assessment Toolkit and the statistical computing software, R (Version 4.3.3).

## Results

### Global burden of AF attributable to high BMI

According to the GBD 2021 database, high BMI emerged as a significant risk factor for AF. Specifically, in 2021, the global burden of high BMI-related AF amounted to 27,236.8 (95% UI: 11,746.5–46,605) deaths and 724,573.6 (95% UI: 303,525.3-1,246,374.3) DALYs. The corresponding ASDR and ASRDALYs were 0.3 per 100,000 persons (95% UI: 0.2–0.6) and 8.7 per 100,000 persons (95% UI: 3.6–15.1), respectively (Table 1).

**Fig. 1:**
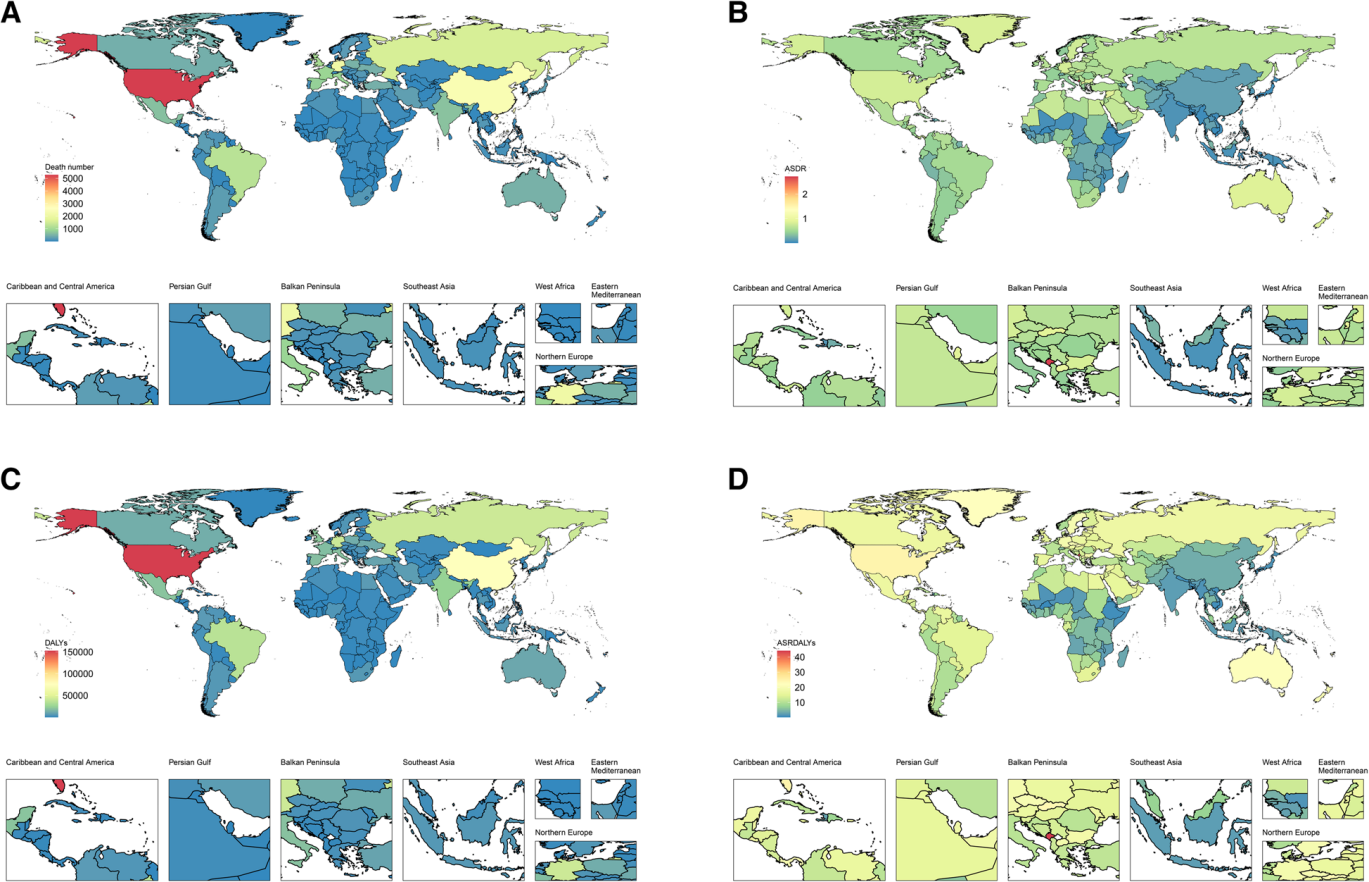
Global burden of high BMI-related AF among 204 countries and territories in 2021. **A** Death number and **B** ASDR of AF to high BMI; **C** DALYs and ASRDALYs of AF related to high BMI. Abbreviations: high BMI: high body mass index; AF: atrial fibrillation; DALYs: disability-adjusted life years; ASDR: age-standardized death rate; ASRDLAYs: age-standardized rate of disability-adjusted life years

Among the 204 countries and territories analyzed (Fig. 1), the United States, with its large population, bore a significantly higher burden of deaths (5,287.12) and DALYs (152,715.79) compared to other nations. Montenegro had the highest ASDR for high BMI-related AF at 2.72 per 100,000 persons (95% UI: 1.14–4.89), while Timor-Leste (0.002 per 100,000 persons, 95% UI: -0.004-0.012) had the lowest ASDR. Similarly, Montenegro had the highest ASRDALYs at 44.18 per 100,000 persons (95% UI: 18.82–44.18), and Timor-Leste had the lowest at 0.25 per 100,000 persons (95% UI: 0.002–0.59).

At the GBD regional level, Australasia exhibited the highest ASDR of high BMI-related AF deaths and DALYs, with 0.9 per 100,000 persons (95% UI: 0.4–1.6) and 21.4 per 100,000 persons (95% UI: 9.1–37.7). Conversely, South Asia and High-income Asia Pacific had the lowest rates, with 0.1 per 100,000 persons (95% UI: 0-0.1) and 2.3 per 100,000 persons (95% UI: 0.9–4.1), and 0.1 per 100,000 persons (95% UI: 0-0.1) and 1.8 per 100,000 persons (95% UI: 0.7–3.2), respectively (Table 1). Subgroup analysis by sex revealed similar global distributions of ASDR and ASRDALYs (Figure S1).

**Table 1:** Deaths, DALYs, ASDR, ASRDALYs of AF attributable to high BMI in 1990 and 2021, and their EAPC (1990–2021)

	Deaths (95% UI)		ASDR (95% UI)		DALYs (95% UI)		ASRDALYs (95% UI)		EAPC (95% CI)	
	1990	2021	1990	2021	1990	2021	1990	2021	ASDR	ASRDALYs
Global	5721.8 (2352.4-9911.6)	27236.8 (11746.5-46605)	0.2 (0.1–0.4)	0.3 (0.2–0.6)	175031.6 (67999.2-298165)	724573.6 (303525.3-1246374.3)	5.2 (2-8.8)	8.7 (3.6–15.1)	1.65 (1.6–1.71)	1.64 (1.6–1.69)
Sex										
Male	1528.3 (616.8-2659.2)	9178.3 (3975.7-16049.8)	0.1 (0.1–0.3)	0.3 (0.1–0.5)	63,380 (23549.9-113098.7)	305937.8 (126785.7-525494.1)	4.2 (1.6–7.4)	8.3 (3.4–14.4)	2.53 (2.46–2.61)	2.32 (2.24–2.4)
Female	4193.5 (1752.4-7181.5)	18058.5 (7717-30759.5)	0.2 (0.1–0.4)	0.4 (0.2–0.6)	111651.7 (44456.8-189380.1)	418635.9 (176495.1-716940.3)	5.8 (2.3–9.7)	8.9 (3.8–15.3)	1.37 (1.31–1.43)	1.32 (1.27–1.37)
SDI										
High SDI	3341.7 (1378.7-5954.4)	13,036 (5608.5-22825.5)	0.3 (0.1–0.6)	0.5 (0.2–0.9)	95785.5 (37100.1-167987.5)	324193.2 (134750.2-566662.2)	8.6 (3.4–15.2)	14.2 (6-24.7)	1.5 (1.41–1.6)	1.58 (1.48–1.68)
High-middle SDI	1703.9 (712-2821.5)	7208.8 (3107.9-12447.4)	0.2 (0.1–0.4)	0.4 (0.2–0.7)	53,742 (21649.4-91085.7)	182168.4 (74546.7-317112.1)	6.2 (2.5–10.4)	9.3 (3.8–16.2)	1.47 (1.41–1.53)	1.28 (1.24–1.32)
Middle SDI	436.4 (172.8-749.1)	4907.3 (2165.8-8209.1)	0.1 (0-0.1)	0.2 (0.1–0.4)	16888.8 (6475.4-28280.1)	150960.1 (62576.5-256705.1)	2 (0.8–3.4)	6.2 (2.6–10.4)	3.73 (3.6–3.85)	3.67 (3.59–3.75)
Low-middle SDI	197 (77.1-349.5)	1782 (757.4-2955.2)	0.1 (0-0.1)	0.2 (0.1–0.3)	7015.2 (2798.7-11741.6)	56693.8 (23028.2-93081.2)	1.4 (0.6–2.3)	4.6 (1.8–7.4)	4.03 (3.93–4.14)	3.97 (3.9–4.05)
Low SDI	30.3 (9-62.8)	264.6 (104.7-485.4)	0 (0–0)	0.1 (0-0.1)	1236.1 (415.7-2329.2)	9595.4 (3654.6-16706.5)	0.6 (0.2–1.2)	2.2 (0.8–3.9)	4.49 (4.23–4.75)	4.22 (4.07–4.36)
Region										
Central Europe	569.8 (246.3-977.3)	1461.8 (651.2-2581.5)	0.5 (0.2–0.8)	0.6 (0.3–1.1)	16330.5 (6519.3-28128.3)	36521.2 (15070.3-64445)	11.7 (4.7–20)	15.4 (6.4–27.4)	0.86 (0.62–1.09)	0.85 (0.7-1)
Western Europe	2049.4 (862-3496.7)	7145.5 (2948.7-12840.4)	0.4 (0.1–0.6)	0.6 (0.2-1)	53705.6 (20725.4-90352.6)	148759.8 (58925.4-264448)	9 (3.5–15.2)	13.8 (5.6–24.8)	1.68 (1.58–1.77)	1.42 (1.3–1.53)
Eastern Europe	784.4 (330.9-1304.1)	2282.6 (979.8–3917)	0.4 (0.2–0.6)	0.6 (0.3–1.1)	24533.4 (10150.5-40575.2)	59342.3 (25168.9-103404.4)	9.5 (3.9–15.6)	16.4 (7-28.5)	1.69 (1.52–1.87)	1.78 (1.65–1.92)
Central Asia	60.5 (25.1-101.5)	162.8 (71.3-276.1)	0.2 (0.1–0.3)	0.3 (0.1–0.5)	2558.6 (1018.2-4384.3)	6544.8 (2671.9-11650.3)	6 (2.4–10.2)	9.1 (3.7–16.1)	1.47 (1.24–1.7)	1.24 (1.16–1.33)
East Asia	58.3 (12.6-132.6)	2543.8 (995.4-4445.6)	0 (0–0)	0.2 (0.1–0.3)	2358.1 (578-4981.1)	78958.7 (30814-135779.9)	0.3 (0.1–0.7)	3.9 (1.5–6.7)	7.96 (7.7–8.21)	8.24 (8.07–8.41)
South Asia	30.4 (8.1–66)	823.4 (345.3-1507.3)	0 (0–0)	0.1 (0-0.1)	1577.2 (489.3-3090.9)	30488.4 (11896.1-53295.1)	0.3 (0.1–0.6)	2.3 (0.9–4.1)	8.07 (7.86–8.29)	7.1 (6.88–7.33)
Southeast Asia	21.8 (3.6–46.6)	508.2 (193.5-902.3)	0 (0–0)	0.1 (0-0.2)	1288.5 (366.3-2504.1)	19001.5 (7628.6-32056.2)	0.5 (0.1-1)	3.2 (1.2–5.4)	7.61 (7.32–7.9)	6.2 (5.97–6.43)
High-income Asia Pacific	31.4 (8.6–69.4)	341.5 (121.7-651.7)	0 (0–0)	0.1 (0-0.1)	1255.1 (355.2-2643.9)	8524.3 (3032.4-15689.4)	0.6 (0.2–1.4)	1.8 (0.7–3.2)	2.83 (2.5–3.15)	3.16 (2.9–3.42)
High-income North America	1240.2 (503.3–2412)	5731.7 (2425.1-9903.7)	0.3 (0.1–0.7)	0.8 (0.3–1.3)	42432.1 (16693.1-79112.6)	165663.6 (70084.1-282995.2)	11.8 (4.6–22)	23.8 (10.1–40.7)	2.5 (2.35–2.66)	2.23 (2.07–2.38)
Caribbean	37.9 (16-61.8)	221.7 (98.8-362.5)	0.2 (0.1–0.3)	0.4 (0.2–0.6)	1268.2 (524.1-2160.2)	5958.3 (2399.4-10212.1)	5.2 (2.2–8.9)	10.9 (4.4–18.7)	2.42 (2.32–2.52)	2.38 (2.31–2.45)
Tropical Latin America	115.5 (43.6–208)	1265.8 (508.7-2201.5)	0.2 (0.1–0.3)	0.5 (0.2–0.9)	5157.5 (1986.6-9372.1)	36494.6 (14652.5-64473.6)	6.2 (2.4–11.3)	14.6 (5.9–25.9)	3.45 (3.19–3.7)	2.8 (2.65–2.95)
Central Latin America	156.1 (63.1-285.6)	1243.7 (550.6-2211.4)	0.3 (0.1–0.5)	0.5 (0.2-1)	5617.7 (2198.9-10138)	36267.5 (15389.5-64769.1)	7.5 (2.9–13.5)	15 (6.4–26.7)	2.39 (2.3–2.49)	2.21 (2.17–2.25)
Andean Latin America	20.3 (7.5–38.4)	182.1 (72.7-329.7)	0.1 (0-0.2)	0.3 (0.1–0.6)	813.4 (299.6-1536.6)	5973.2 (2405.4-10708.1)	4.1 (1.5–7.6)	10.4 (4.2–18.5)	3.31 (3.15–3.48)	2.99 (2.91–3.06)
Southern Latin America	96.4 (39.7-177.4)	416.6 (175.5-757.8)	0.3 (0.1–0.5)	0.4 (0.2–0.8)	2900.8 (1135.5–5268)	9416.4 (3834.2-16720.6)	6.7 (2.6–12)	10.4 (4.3–18.5)	2.65 (2.23–3.07)	1.89 (1.64–2.15)
Oceania	3 (1.2–6.1)	13.7 (5.9–25)	0.2 (0.1–0.4)	0.3 (0.1–0.5)	134.2 (51.9-251.1)	571.9 (237.3-1010.9)	4.8 (1.8–9.5)	8.1 (3.4–14.2)	1.52 (1.44–1.59)	1.65 (1.59–1.71)
Australasia	97.1 (38.2-182.3)	563.2 (233.2-1041.1)	0.5 (0.2–0.9)	0.9 (0.4–1.6)	2627.5 (1015.6–4866)	12414.3 (5303.8-21746.9)	11.5 (4.5–21.4)	21.4 (9.1–37.7)	2.13 (1.89–2.38)	2.13 (1.98–2.28)
Central Sub-Saharan Africa	5.1 (1.7–10.8)	67.3 (24.7–136)	0 (0-0.1)	0.2 (0.1–0.4)	197.9 (67.8-389.5)	2219.9 (807-4057.9)	1 (0.3-2)	5.1 (1.9–9.3)	5.88 (5.69–6.06)	5.33 (5.22–5.44)
Eastern Sub-Saharan Africa	8.3 (2.2–17.8)	90.6 (34.2-171.2)	0 (0–0)	0.1 (0-0.2)	391.8 (121.3-790.1)	3725.9 (1473.6-6591.3)	0.6 (0.2–1.1)	2.5 (1-4.4)	5.25 (5.13–5.36)	4.85 (4.79–4.91)
North Africa and Middle East	249.2 (99.2-441.4)	1659.6 (702.6-2810.6)	0.2 (0.1–0.4)	0.6 (0.2-1)	7059.4 (2801.4-12195.7)	42,718 (18785.3-73469.5)	5.3 (2.1–9.2)	11.7 (5.1–20.2)	2.91 (2.68–3.15)	2.58 (2.44–2.71)
Southern Sub-Saharan Africa	36.5 (15.9–63)	206.7 (94.3-342.5)	0.2 (0.1–0.3)	0.6 (0.3–0.9)	1444.3 (597.2-2409.1)	6615 (2824.5-11344.5)	6 (2.5–10.1)	13.8 (5.9–23.5)	3.51 (3.11–3.91)	2.72 (2.5–2.95)
Western Sub-Saharan Africa	50.1 (18.4–92.7)	304.5 (127-522.1)	0.1 (0-0.2)	0.3 (0.1–0.5)	1379.6 (541.7-2412.4)	8393.9 (3406.4-14015.1)	2 (0.8–3.7)	5.9 (2.4–9.8)	3.08 (2.92–3.23)	3.2 (3.1–3.3)

*Abbreviations*: *AF* Atrial fibrillation, *high BMI* High body mass index, *EAPC* Estimated annual percentage change, *ASDR* Age standardized death rate, *DALYs* Disability-adjusted life years, *ASRDLAYs* Age-standardized rate of disability-adjusted life years, *SDI* Sociodemographic index, *UI* Uncertainty interval, *CI* Confidence interval

### Temporal trends of AF burden attributable to high BMI from 1990 to 2021

**Fig. 2:**
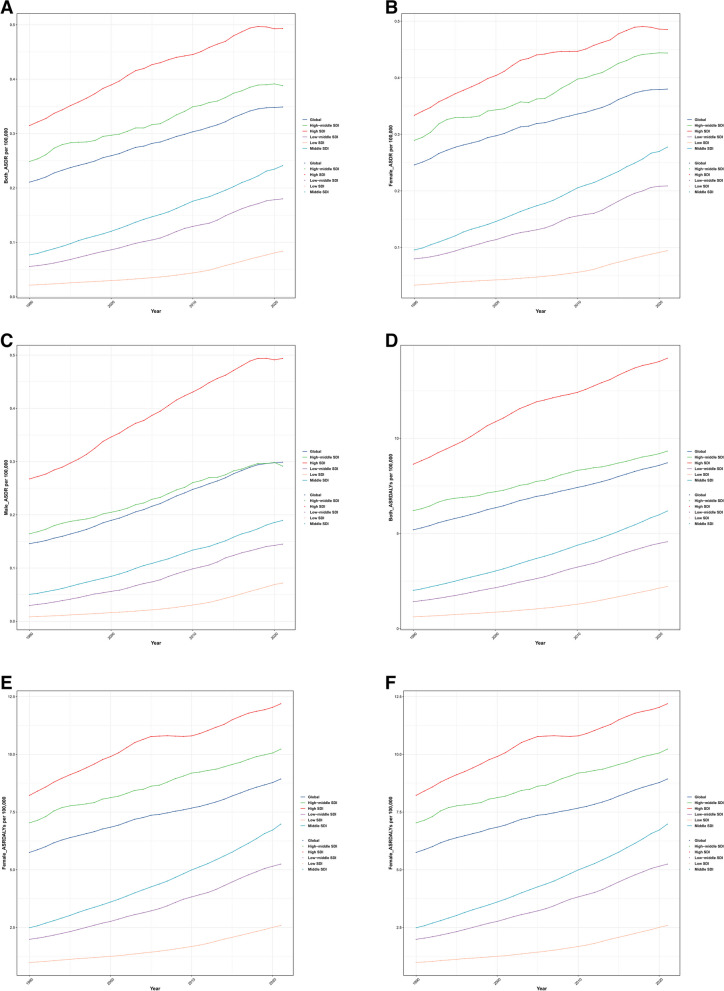
Temporal trends of age-standardized AF burden attributable to APMP2.5 in territories with low to high SDIs from 1990 to 2021. **A**, **B** ASDR and ASRDALYs of AF; **C**, **D** ASDR and ASRDALYs of AF related to high BMI in females; **E**, **F** ASDR and ASRDALYs of AF related to high BMI in males. Abbreviations: AF: atrial fibrillation; high BMI: high body mass index; SDI: sociodemographic index; ASDR: age-standardized death rate; ASRDLAYs: age-standardized rate of disability-adjusted life years

Over the past three decades, the global burden of AF attributable to high BMI has grown substantially. In 2021, the number of deaths and DALYs caused by high BMI-related AF reached 27,000 (95% UI: 12,000–47,000) and 725,000 (95% UI: 304,000–1,250,000), respectively (Figure S2 A-B). However, after adjusting for population growth and aging, the ASDR and ASRDALYs remained relatively stable at 0.3 per 100,000 persons (EAPC: 1.65 [95% CI: 1.6–1.71]) and 8.7 per 100,000 persons (EAPC: 1.64 [95% CI: 1.6–1.69]), respectively. Notably, despite the overall stability, both ASDR and ASRDALYs exhibited a continuous increasing trend throughout the study period (Figure S2 C-D). Further analysis revealed marked disparities in the AF burden caused by high BMI across different SDI regions (Fig. 2A-B). From 1990 to 2021, high SDI regions consistently had the highest ASDR and ASRDALYs in 2021. In contrast, low SDI regions had the lowest ASDR and ASRDALYs since 1990.

### Regional burden and trends of AF attributable to high BMI

**Fig. 3:**
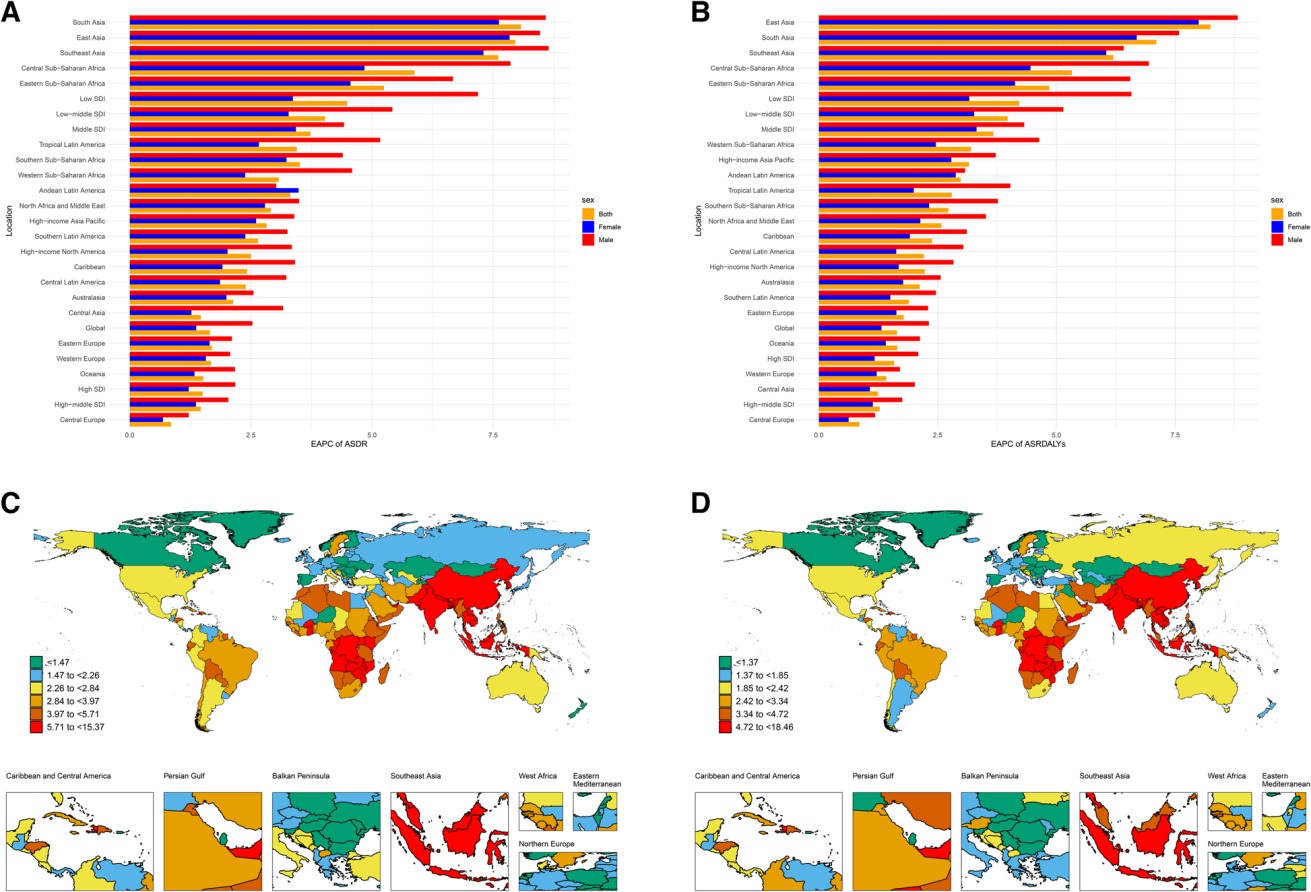
EAPC of ASDR and ASRDALYs attributable to high BMI-related AF from 1990 to 2021. **A**, **B** EAPC of ASDR and ASRDALYs in different SDI quintiles and regions **C**, **D** EAPC of ASDR and ASRDALYs in 204 countries and territories. Abbreviations: EAPC: estimated annual percentage change; AF: atrial fibrillation; high BMI: high body mass index; ASDR: age-standardized death rate; ASRDLAYs: age-standardized rate of disability adjusted life years

 To further elucidate the regional disparities in the burden of AF related to high BMI, we analyzed the ASDR and ASRDALYs based on region-specific data (Fig. 3A-B). The high-income Central Europe region had the lowest EAPC for ASDR at 0.86 (95% CI: 0.62–1.09), suggesting the slowest increase in the mortality rate of AF caused by high BMI. Conversely, South Asia (EAPC = 8.07, 95% CI: 7.86–8.29), East Asia (EAPC = 7.96, 95% CI: 7.7–8.21), and Southeast Asia (EAPC = 7.61, 95% CI: 7.32–7.9) had the fastest ASDR growth rates.

At the country level (Fig. 3C), Guam (EAPC=-1.16, 95% CI: -1.63, -0.69) and Finland (EAPC=-0.57, 95% CI: -0.95, -0.8) had the largest decreases in ASDR. However, some countries exhibited relatively rapid growth trends in ASDR, with Bangladesh (EAPC = 15.36919699, 95% CI: 14.142–16.609), Indonesia (EAPC = 12.26148542, 95% CI: 11.522–13.006), and Nepal (EAPC = 11.22605849, 95% CI: 11.052–11.401) showing the most significant increases.

### Sex-specific AF burden and trends attributable to high BMI

Throughout the study period, the burden of high BMI-related AF was consistently higher in females than in males, but the gender gap gradually narrowed over time. In females, the ASDR increased from 0.2 per 100,000 persons (95% UI: 0.1–0.4) in 1990 to 0.4 per 100,000 persons (95% UI: 0.2–0.6) in 2021, while the ASRDALYs increased from 5.8 to 8.9 per 100,000 persons (Table 1). Globally, the ASDR and ASRDALYs of high BMI-related AF in males increased substantially over the past few decades, from 0.1 per 100,000 persons (95% UI: 0.1–0.3) and 4.2 per 100,000 persons (95% UI: 1.6–7.4) in 1990 to 0.3 per 100,000 persons (95% UI: 0.1–0.5) and 8.3 per 100,000 persons (95% UI: 3.4–14.4) in 2021, respectively.

**Fig. 4:**
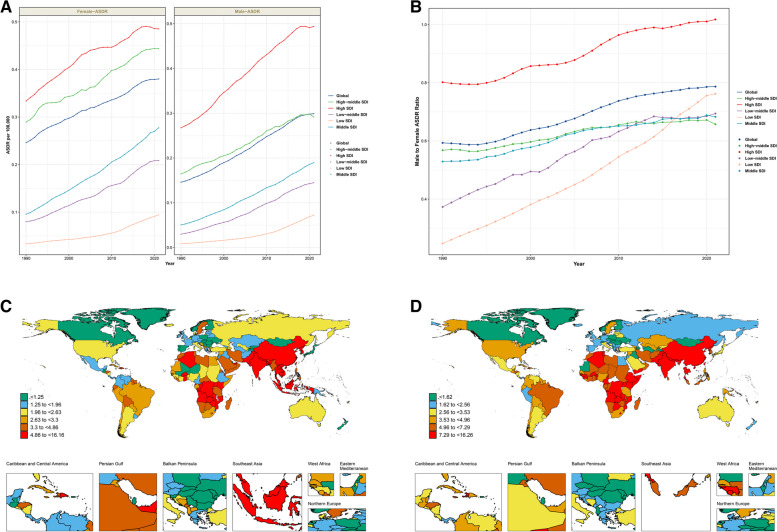
Sex differences and trends in high BMI -related AF in different regions from 1990 to 2021. **A** The ASDR trends with low to high SDIs in female and male, respectively; **B** Male-to-female ratios of ASDR in five SDI regions. **C**, **D** EAPC of ASDR in 204 countries and territories in female and male. Abbreviations: high BMI: high body mass index; AF: atrial fibrillation; SDI: sociodemographic index; EAPC: estimated annual percentage change; ASDR: age-standardized death rate

 Regional analysis showed that the ASDR and ASRDALYs for both males and females in all SDI regions exhibited an increasing trend from 1990 to 2021 (Fig. 4A). In 2021, the Australasia region had the highest ASDR for both males (0.8 per 100,000 persons, 95% UI: 0.3–1.5) and females (0.9 per 100,000 persons, 95% UI: 0.4–1.6), while the high-income Asia Pacific region had the lowest ASDR for both sexes (males: 0.0 per 100,000 persons, 95% UI: 0-0.1; females: 0.1 per 100,000 persons, 95% UI: 0-0.1) (Tables S1-2). However, the ratio of male to female ASDR in high SDI regions was much higher than in other regions (Fig. 4B), indicating the most significant gender differences in this region. The ASDR growth rate was highest for males in the high-income Southeast Asia region (EAPC = 8.64, 95% CI: 8.08–9.22) and for females in the East Asia region (EAPC = 7.84, 95% CI: 7.6–8.07) (Tables S1-2).

Country-level analysis (Fig. 4C-D) revealed that Montenegro had the highest ASDR and ASRDALYs for males, while the United Arab Emirates had the highest for females.

### Age-specific AF burden and trends attributable to high BMI

**Fig. 5:**
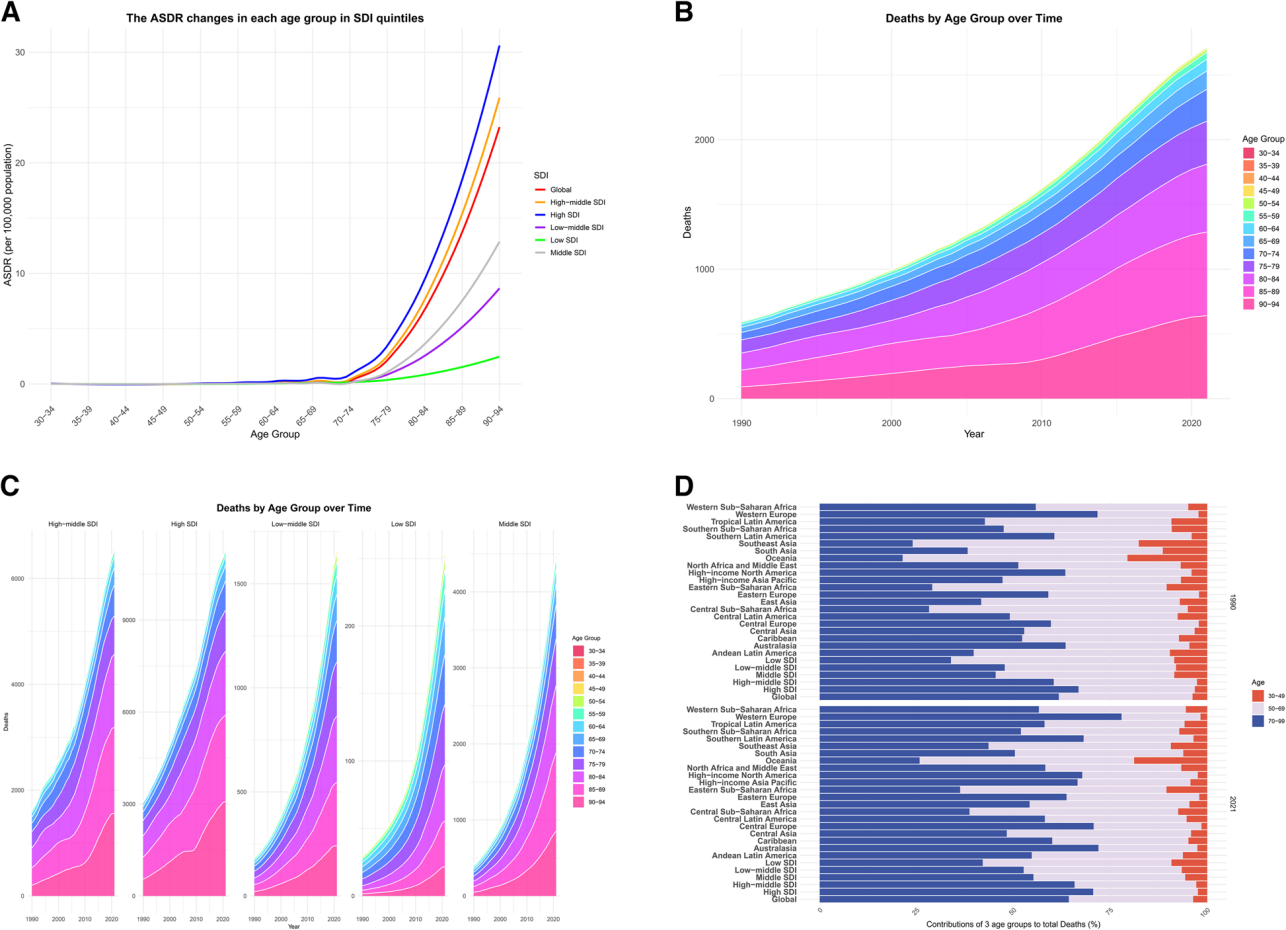
AF burden attributable to high BMI and the trends by age groups in different regions. **A** The ASDR changes in each age group (30–95 + years, 5-year intervals) in SDI quintiles; **B**, **C** The trends of deaths in each age group from 1990 to 2021 with high to low SDI regions; **D** The three age groups as proportions of total deaths globally. Abbreviations: high BMI: high body mass index; AF: atrial fibrillation; ASDR: age-standardized death rate; SDI: sociodemographic index

 In the age subgroup analysis, the ASDR and ASDALYs of high BMI-related AF increased sharply with age in all SDI regions (Fig. 5A, Figure S4A), peaking in the population aged 95 and above.

As shown in Fig. 5B, the number of deaths increased substantially in all age groups from 1990 to 2021, with the elderly population, particularly those aged 70 and above, accounting for the majority of deaths. Moreover, the number of deaths increased more rapidly in high SDI regions compared to low-middle SDI regions, with high SDI regions having the highest number of deaths and DALYs (Fig. 5, Figure S4).

For further analysis, we divided the population into three age groups: 30–49 years (young), 50–69 years (middle-aged), and 70–89 years (elderly). The proportion of the elderly population in the total number of deaths reached 87.80% in 2021, a slight increase from the previous year. We then calculated and compared the percentage changes in the number of deaths and DALYs for each SDI region in 1990 and 2021 (Fig. 5D, Figure S4D). The proportion of deaths among the young and middle-aged population due to high BMI-related AF was higher in low SDI regions than in high SDI regions. Interestingly, Central Asia was the only region among the 27 regions where the proportion of the elderly population decreased from 77.6% in 1990 to 75.7% in 2021 (Fig. 5).

## Discussion

The results indicate a significant global increase in absolute deaths and DALYs from high BMI-related AF, with slight rises in corresponding ASRs. Females consistently bore a higher disease burden. Regional variations were observed: high SDI regions showed the highest burden, while low-income countries experienced rapid increases.

Globally and nationally, trends in ASDR and ASRDALYs for high BMI-related AF mirror overall AF burden trends. This correlation likely stems from shared risk factors between obesity and AF, including hypertension, cardiac abnormalities, obstructive sleep apnea and metabolic syndrome [6, 8, 17, 18]. Despite stable global ASDR and ASRDALYs for high BMI-related AF, significant disparities exist across SDI regions. These findings underscore the importance of considering socioeconomic factors in AF prevention strategies to address regional health disparities. Central Europe showed slower increases in high BMI-related AF mortality, possibly due to established obesity prevention policies. Southeast Asia experienced the fastest growth rates, potentially linked to rapid urbanization and lifestyle changes [19].

At the country level, Guam and Finland had significant declines in high BMI-related AF mortality, while Bangladesh, Indonesia, and Nepal saw rapid increases, highlighting challenges in developing countries. Globally, compared to males, females had almost twice the ASDR and ASRDALYs in 1990, especially in Gulf countries, which may be related to factors such as lifestyle changes and reduced physical activity [20]. A meta-analysis showed that high BMI had a greater impact on the risk of AF in females than in males (RR: 1.55 vs. 1.37) [18]. Another study also found that abdominal obesity and metabolic syndrome were more closely related to AF in females [21]. Moreover, studies have shown that female patients with AF have a higher risk of thromboembolism events, such as stroke, compared to males [22]. This may be related to factors such as changes in coagulation function and hormone levels in females [23].Moreover, there are gender differences in the diagnosis and treatment of female patients with AF [24]. Females also have a longer life expectancy than males, and the incidence of AF increases with age.

Our study shows that people aged 70 and above account for the majority of the high BMI-related AF burden, consistent with previous evidence on age-related susceptibility to high BMI and AF. Epidemiological surveys have revealed that the obesity rate among the elderly is growing rapidly in many countries [25]. A meta-analysis that included 5 cohort studies showed that the association between BMI and the risk of AF onset was stronger in the elderly population compared to the young and middle-aged (RR: 1.54 vs. 1.33) [18].

In light of these compelling results, we propose a multifaceted approach to address this burgeoning health concern. Primarily, it is imperative to direct concerted efforts towards rapidly developing regions and the elderly demographic, as these populations are disproportionately affected. For example, Japan implemented the “Specific Health Checkups and Specific Health Guidance” system in 2008, aimed at reducing the obesity rate among adults, which has led to a continuous reduction in the high BMI-related AF burden [26]. Furthermore, the formulation of prevention and control measures must take into account the nuanced gender differences observed in our study, ensuring that interventions are tailored to address the unique needs of both male and female populations. Healthcare providers could strengthen screening and early intervention for high-risk populations, such as females and the elderly, while improving their disease awareness. Secondly, health policymakers might consider developing targeted prevention and control measures based on local epidemiological characteristics, allocating medical resources accordingly. On the one hand, females are more prone to obesity-related cardiovascular diseases, including AF [27]. On the other hand, the obesity rate among males has risen rapidly in recent years, and the associated AF risk cannot be ignored [28]. Additionally, clinicians could offer individualized weight reduction guidance and integrate multidisciplinary efforts to provide comprehensive health management services for patients with AF and high BMI. By translating this research evidence into targeted patient education materials and personalized management strategies, high-risk individuals may be better equipped to reduce the incidence of obesity-related AF and improve their prognosis.

Although this GBD study comprehensively assessed the global burden of high BMI-related AF, our study still has some limitations. First, because BMI data are mainly derived from demographic surveys and health examinations, measurement errors cannot be completely avoided, which may lead to misclassification of individual exposures [29]. In addition, previous studies have reported that the correlation between different types of obesity (such as abdominal obesity, obesity-related metabolic syndrome, etc.) and the risk of AF may be different, but GBD 2021 did not consider these factors [17]. Atrial fibrillation itself rarely directly causes death, which may introduce implicit bias in its association with mortality, potentially leading to under- or overestimation of its impact. The GBD 2021 dataset specifically provides data on the overall burden of AF caused by BMI exposure as a risk factor, but it does not distinguish between subtypes such as paroxysmal AF and persistent AF. In fact, the impact of obesity on different types of AF may differ, which requires further differentiation in future studies [30]. Finally, due to the limitations of primary care diagnostic conditions and insufficient awareness of AF, the phenomenon of underdiagnosis of AF may be more common in primary care and underdeveloped regions [31, 32]. Individual patient baseline clinical variables and imaging data were not included in the analysis, potentially introducing confounding factors that could provide alternative explanations for our observations.While we observed regional, age, and sex differences in trends, our explanations for these differences are largely speculative. We acknowledge that these interpretations should be considered hypotheses rather than definitive conclusions.

## Conclusion

In conclusion, our study provides evidence on the latest burden of AF caused by high BMI exposure. Over the past three decades, the absolute number of deaths and DALYs from AF due to high BMI has increased substantially worldwide, owing to population growth, aging, and the obesity epidemic. Although the burden of AF associated with high BMI exhibits gender differences in ASDR and ASDALYs and varies with SDI, the overall impact of high BMI on the total burden of AF shows an upward trend. Specifically, females, the elderly, and high SDI regions bear a heavier burden of high BMI-related AF. This is consistent with previous research findings on the gender differences in the impact of obesity on AF, the prevalence of AF in the elderly, and the burden of obesity in high-income countries. This suggests that when formulating prevention and control strategies, gender and regional differences should be considered, and more attention should be given to vulnerable populations.

## Supplementary Information

Supplementary Material 1: Table S1. Deaths, ASDR, ASRDALYs of AF attributable to high BMI in 1990 and 2021, and their EAPC (1990-2021) in male. Table S2. Deaths, ASDR, ASRDALYs of AF attributable to high BMI in 1990 and 2021, and their EAPC (1990-2021) in female. Table S3. The death cases and ASDR of AF burden attributable to high BMI of each age group (30-95+ years, 5-year intervals) in 1990 and 2021. Table S4. The death number of three age groups in different regions from 1990-2021. Fig.S1 Global burden of high BMI-related AF among 204 countries and territories by sex in 2021, related to Fig. 1. Fig. S2 Temporal trends of AF burden attributable to high BMI by gender from 1990 to 2021, related to Fig. 2. Fig. S3 Sex differences and trends in high BMI-related AF in different regions from 1990 to 2021, related to Fig. 4. Fig. S4 AF burden attributable to high BMI and the trends by age groups in different regions, related to Fig. 5.

## Data Availability

GBD study 2021 data resources were available online from the Global Health Data Exchange (GHDx) query tool (http://ghdx.healthdata.org/gbd-results-tool).

## Abbreviations

AF: Atrial Fibrillation
BMI: Body Mass Index
GBD: Global Burden of Disease
DALYs: Disability-Adjusted Life Years
EAPC: Estimated Annual Percentage Change
SDI: Socio-Demographic Index
ASDR: Age-Standardized Death Rate
ASR: Age-Standardized Rate
ASRDALYs: Age-Standardized DALY Rates
UI: Uncertainty Interval
GATHER: Guidelines for Accurate and Transparent Health Estimates Reporting
YLLs: Years of Life Lost
YLDs: Years Lived with Disability
CI: Confidence Interval
GHDx: Global Health Data Exchange
IHME: Institute for Health Metrics and Evaluation
RR: Relative Risk

## Data availability

No datasets were generated or analysed during the current study.
