# Supplementary material for: Global burden of atrial fibrillation attributable to high body mass index from 1990 to 2021: findings from the Global Burden of Disease Study 2021
Source: BMC Cardiovasc Disord. 2024 Oct 8;24:542. doi: 10.1186/s12872-024-04202-5 (PMC11459850; doi:10.1186/s12872-024-04202-5)
Supplement: Supplementary file 1 — Supplementary Material 1: Table S1. Deaths, ASDR, ASRDALYs of AF attributable to high BMI in 1990 and 2021, and their EAPC (1990-2021) in male. Table S2. Deaths, ASDR, ASRDALYs of AF attributable to high BMI in 1990 and 2021, and their EAPC (1990-2021) in female. Table S3. The death cases and ASDR of AF burden attributable to high BMI of each age group (30-95+ years, 5-year intervals) in 1990 and 2021. Table S4. The death number of three age groups in different regions from 1990-2021. Fig.S1 Global burden of high BMI-related AF among 204 countries and territories by sex in 2021, related to Fig. 1. Fig. S2 Temporal trends of AF burden attributable to high BMI by gender from 1990 to 2021, related to Fig. 2. Fig. S3 Sex differences and trends in high BMI-related AF in different regions from 1990 to 2021, related to Fig. 4. Fig. S4 AF burden attributable to high BMI and the trends by age groups in different regions, related to Fig. 5. [file 12872_2024_4202_MOESM1_ESM.zip › table and title.docx]

**Table S1.** Deaths, ASDR, ASRDALYs of AF attributable to high BMI in 1990 and 2021, and their EAPC (1990-2021) in male.

**Table S2.** The death cases and ASDR of AF burden attributable to high BMI in 1990

and 2021, and their EAPC from 1990 to 2021 in female.

**Table S3.** The death cases and ASDR of AF burden attributable to high BMI of each

age group (30-95+ years, 5-year intervals) in 1990 and 2021.

**Table S4.** The death number of three age groups in different regions from 1990-2021.

**Fig.S1** Global burden of high BMI-related AF among 204 countries and territories by

sex in 2021, related to Fig.1.

**Fig.S2** Temporal trends of AF burden attributable to high BMI by gender from 1990 to 2021, related to Fig.2.

**Fig.S3** Sex differences and trends in high BMI-related AF in different regions from

1990 to 2021, related to Fig.4.

**Fig.S4** AF burden attributable to high BMI and the trends by age groups in different

regions, related to Fig.5.

| **Table S1.** Deaths, ASDR, ASRDALYs of AF attributable to high BMI in 1990 and 2021, and their EAPC (1990-2021) in male. | | | | | | | | | | |
| --- | --- | --- | --- | --- | --- | --- | --- | --- | --- | --- |
|  | Deaths (95% UI) | | ASDR (95% UI) | | DALYs (95% UI) | | ASRDALYs (95% UI) | | EAPC (95% CI) | |
|  | 1990 | 2021 | 1990 | 2021 | 1990 | 2021 | 1990 | 2021 | ASDR | ASRDALYs |
| Global | 1528.3 (616.8-2659.2) | 9178.3 (3975.7-16049.8) | 0.1 (0.1-0.3) | 0.3 (0.1-0.5) | 63380 (23549.9-113098.7) | 305937.8 (126785.7-525494.1) | 4.2 (1.6-7.4) | 8.3 (3.4-14.4) | 2.53 (2.46-2.61) | 2.32 (2.24-2.4) |
| SDI | | | | | | | | | | |
| High SDI | 961.9 (385-1735.1) | 4904.1 (2095.3-8901.6) | 0.3 (0.1-0.5) | 0.5 (0.2-0.9) | 38633 (14652.9-72031) | 157507.6 (65285.7-276084.1) | 8.8 (3.3-16.3) | 16.3 (6.8-28.5) | 2.18 (2.07-2.29) | 2.09 (1.97-2.22) |
| High-middle SDI | 385 (156.5-650.4) | 1963.7 (817.7-3506.1) | 0.2 (0.1-0.3) | 0.3 (0.1-0.5) | 16506.5 (6311.1-28673.5) | 64832.3 (26305.4-114448.9) | 4.7 (1.8-8) | 7.9 (3.2-14) | 2.04 (1.96-2.12) | 1.76 (1.7-1.81) |
| Middle SDI | 122 (45.9-210.4) | 1577.4 (688.7-2631.1) | 0.1 (0-0.1) | 0.2 (0.1-0.3) | 5734.7 (2156.5-9740.6) | 57703 (23358.4-98359.3) | 1.4 (0.5-2.5) | 5.2 (2.1-8.8) | 4.42 (4.26-4.59) | 4.32 (4.19-4.46) |
| Low-middle SDI | 49.8 (17.8-89.4) | 617.4 (262.2-1015.8) | 0 (0-0.1) | 0.1 (0.1-0.2) | 2109.3 (806.3-3670.9) | 21792.5 (8562.5-35919.5) | 0.8 (0.3-1.5) | 3.8 (1.5-6.2) | 5.42 (5.21-5.63) | 5.15 (4.97-5.33) |
| Low SDI | 6.1 (0.8-15) | 103.4 (36.5-190.3) | 0 (0-0) | 0.1 (0-0.1) | 259.3 (54.3-607) | 3714.1 (1309.5-6653.6) | 0.3 (0-0.6) | 1.8 (0.6-3.3) | 7.19 (6.99-7.38) | 6.58 (6.5-6.66) |
| Region | | | | | | | | | | |
| Central Europe | 168.6 (67.9-300.2) | 459.4 (195.2-858.6) | 0.4 (0.2-0.7) | 0.5 (0.2-1) | 6260.8 (2485.4-11572.6) | 14671.5 (5957.9-27155.6) | 10.9 (4.2-20) | 15.7 (6.4-28.9) | 1.22 (1.01-1.42) | 1.18 (1.04-1.32) |
| Western Europe | 550.3 (233.6-939.6) | 514.9 (218.2-910) | 0.3 (0.1-0.4) | 0.5 (0.2-0.9) | 20412.3 (7849.2-37504.8) | 65400.8 (25837.5-122159.5) | 9 (3.5-16.3) | 15.1 (6-28.1) | 2.11 (1.92-2.3) | 1.71 (1.58-1.83) |
| Eastern Europe | 129.9 (51.4-228.2) | 514.9 (218.2-910) | 0.3 (0.1-0.4) | 0.5 (0.2-0.9) | 5795.9 (2240-9761.3) | 17704.8 (7058.2-32274.2) | 7.2 (2.8-12.2) | 14.3 (5.7-25.8) | 2.11 (1.92-2.3) | 2.3 (2.15-2.45) |
| Central Asia | 9.5 (4-16.6) | 40.6 (16.7-72.2) | 0.1 (0-0.1) | 0.2 (0.1-0.3) | 715.7 (283.9-1237.7) | 2444.7 (961.7-4510.6) | 4 (1.6-6.8) | 7.5 (2.9-13.2) | 3.17 (2.97-3.36) | 2.02 (1.94-2.11) |
| East Asia | 13.5 (1.9-33.2) | 635.4 (249.8-1142.1) | 0 (0-0) | 0.1 (0-0.2) | 753.5 (152.1-1740.3) | 26751.5 (9924.1-46469.7) | 0.2 (0-0.5) | 2.9 (1.1-4.9) | 8.47 (8.14-8.8) | 8.82 (8.59-9.04) |
| South Asia | 10.6 (1.8-24.5) | 301.3 (106.8-570) | 0 (0-0) | 0.1 (0-0.1 | 553.1 (142.3-1208.3) | 11580 (4446-20652.3) | 0.2 (0-0.5) | 1.8 (0.7-3.2) | 8.58 (8.34-8.83) | 7.58 (7.31-7.85) |
| Southeast Asia | 3.6 (-0.5-9.8) | 94.1 (27.7-178.7) | 0 (0-0) | 0 (0-0.1) | 315.4 (51.2-704.9) | 4839.8 (1729.8-8880) | 0.2 (0-0.6) | 1.7 (0.6-3.1) | 8.64 (8.08-9.22) | 6.42 (6.1-6.73) |
| High-income Asia Pacific | 9 (1.3-20.7) | 106.3 (34.9-234.3) | 0 (0-0) | 0 (0-0.1) | 478 (115.7-1003.6) | 3533.2 (1162.6-7138.1) | 0.5 (0.1-1.2) | 1.8 (0.6-3.6) | 3.39 (3.03-3.76) | 3.72 (3.58-3.87) |
| High-income North America | 397.3 (156.8-788.5) | 2507.9 (1063-4333.8) | 0.3 (0.1-0.6) | 0.8 (0.4-1.5) | 18342.2 (6873.7-35802.8) | 87785.1 (37036.3-151748.3) | 12.7 (4.8-24.4) | 29.1 (12.3-50.3) | 3.35 (3.18-3.51) | 2.83 (2.66-3.01) |
| Caribbean | 11.2 (4.3-18.8) | 77.1 (32.1-132.6) | 0.1 (0-0.2) | 0.3 (0.1-0.6) | 443.8 (178-745.8) | 2395.2 (968.8-4117) | 3.8 (1.5-6.3) | 9.6 (3.9-16.6) | 3.41 (3.3-3.52) | 3.11 (3.03-3.2) |
| Tropical Latin America | 31.8 (11.2-59.8) | 461 (186.7-811.8) | 0.1 (0-0.2) | 0.5 (0.2-0.9) | 1886 (666.7-3571) | 16077.6 (6312.1-28359.7) | 4.7 (1.6-8.7) | 14.9 (5.9-26.3) | 5.17 (4.85-5.49) | 4.03 (3.8-4.27) |
| Central Latin America | 48 (18.6-89.6) | 465.9 (203-859.5) | 0.2 (0.1-0.3) | 0.5 (0.2-0.9) | 2006 (763.5-3691.1) | 15601.7 (6518.6-28035.5) | 5.5 (2.1-10) | 14.3 (6-25.5) | 3.23 (3.07-3.4) | 3.04 (2.96-3.12) |
| Andean Latin America | 9 (3.3-18.1) | 71.6 (30.3-134.7) | 0.1 (0-0.2) | 0.3 (0.1-0.5) | 359.5 (129.4-687.9) | 2683.1 (1104.2-4887.6) | 3.8 (1.4-7.2) | 9.9 (4.1-18.1) | 3.02 (2.86-3.18) | 3.08 (2.98-3.17) |
| Southern Latin America | 27.7 (10.8-50.3) | 136.7 (54.2-252.3) | 0.2 (0.1-0.3) | 0.4 (0.2-0.7) | 1101.8 (419.8-1971.9) | 4096.5 (1624.6-7378.3) | 5.8 (2.2-10.4) | 11 (4.4-19.8) | 3.26 (2.84-3.67) | 2.47 (2.22-2.72) |
| Oceania | 1 (0.4-2.1) | 5.3 (2.2-9.6) | 0.1 (0-0.2) | 0.2 (0.1-0.4) | 50 (18.1-96.6) | 242.6 (100.5-434.8) | 3.3 (1.2-6.7) | 6.4 (2.6-11.3) | 2.17 (2.05-2.3) | 2.13 (2.06-2.2) |
| Australasia | 28.8 (10.8-54.7) | 214.2 (86.1-400.5) | 0.4 (0.1-0.8) | 0.8 (0.3-1.5) | 1102.4 (421.3-2082.7) | 5987.5 (2478.2-10852.5) | 11.5 (4.3-21.6) | 23.5 (9.8-43) | 2.55 (2.28-2.83) | 2.56 (2.39-2.74) |
| Central Sub-Saharan Africa | 1.5 (0.2-3.7) | 26.4 (8.5-52) | 0 (0-0.1) | 0.2 (0.1-0.4) | 58.3 (12.1-138.7) | 927 (330.6-1720.3) | 0.6 (0.1-1.5) | 5.1 (1.8-9.7) | 7.86 (7.57-8.15) | 6.95 (6.74-7.15) |
| Eastern Sub-Saharan Africa | 1.8 (0.1-5.1 | 31.5 (10.6-62.2) | 0 (0-0) | 0.1 (0-0.1) | 72.8 (10.1-191.8) | 1214.9 (435.5-2261.3) | 0.2 (0-0.6) | 1.7 (0.6-3.3) | 6.67 (6.51-6.84) | 6.56 (6.42-6.69) |
| North Africa and Middle East | 55.6 (20-105.5) | 515 (223.5-898.6) | 0.1 (0-0.2) | 0.3 (0.1-0.6) | 1986.6 (759.6-3528.5) | 16910.7 (7276-29083) | 2.9 (1.1-5.1) | 9 (3.8-15.3) | 3.5 (3.39-3.61) | 3.52 (3.45-3.59) |
| Southern Sub-Saharan Africa | 7.8 (3.2-14.7) | 50.1 (20.5-84.1) | 0.1 (0-0.2) | 0.4 (0.2-0.7) | 333.7 (129.1-569.5) | 1972 (772.8-3338.4) | 3.4 (1.3-6) | 10.5 (4.1-17.8) | 4.4 (4.01-4.78) | 3.77 (3.5-4.04) |
| Western Sub-Saharan Africa | 11.9 (3.9-23.3) | 117.8 (45.2-210.8) | 0.1 (0-0.1) | 0.3 (0.1-0.5) | 352.1 (116.7-682.7) | 3117.6 (1218-5301.5) | 1.2 (0.4-2.3) | 4.9 (1.9-8.5) | 4.59 (4.5-4.68) | 4.64 (4.54-4.73) |
| Abbreviations: AF: atrial fibrillation; high BMI: high body mass index; EAPC: estimated annual percentage change; ASDR: age standardized death rate; DALYs: disability-adjusted life years; ASRDLAYs: age-standardized rate of disability-adjusted life years; SDI: sociodemographic index; UI: uncertainty interval; CI: confidence interval. | | | | | | | | | | |

| **Table S2.** Deaths, ASDR, ASRDALYs of AF attributable to high BMI in 1990 and 2021, and their EAPC (1990-2021) in female. | | | | | | | | | | |
| --- | --- | --- | --- | --- | --- | --- | --- | --- | --- | --- |
|  | Deaths (95% UI) | | ASDR (95% UI) | | DALYs (95% UI) | | ASRDALYs (95% UI) | | EAPC (95% CI) | |
|  | 1990 | 2021 | 1990 | 2021 | 1990 | 2021 | 1990 | 2021 | ASDR | ASRDALYs |
| AF | 4193.5 (1752.4-7181.5) | 18058.5 (7717-30759.5) | 0.2 (0.1-0.4) | 0.4 (0.2-0.6) | 111651.7 (44456.8-189380.1) | 418635.9 (176495.1-716940.3) | 5.8 (2.3-9.7) | 8.9 (3.8-15.3) | 1.37 (1.31-1.43) | 1.32 (1.27-1.37) |
| SDI | | | | | | | | | | |
| High SDI | 2379.8 (1013-4210.8) | 8131.9 (3434-14114) | 0.3 (0.1-0.6) | 0.5 (0.2-0.8) | 57152.5 (22588.9-98477.3) | 166685.6 (69814.3-283782) | 8.2 (3.3-14.1) | 12.2 (5.1-21) | 1.22 (1.11-1.32) | 1.17 (1.08-1.27) |
| High-middle SDI | 1318.9 (563.7-2211.9) | 5245 (2278.4-8906.2) | 0.3 (0.1-0.5) | 0.4 (0.2-0.8) | 37235.5 (14992.3-64021.4) | 117336.1 (48161.1-202250.5) | 7 (2.8-12) | 10.2 (4.2-17.6) | 1.37 (1.31-1.43) | 1.14 (1.1-1.17) |
| Middle SDI | 314.4 (124.9-542.3) | 3329.9 (1452.2-5594) | 0.1 (0-0.2) | 0.3 (0.1-0.5) | 11154.1 (4240.4-18736.7) | 93257.1 (39029.6-158221.2) | 2.5 (0.9-4.2) | 7 (2.9-11.8) | 3.43 (3.32-3.54) | 3.32 (3.26-3.37) |
| Low-middle SDI | 147.2 (58.1-259.4) | 1164.5 (497.8-1926.8) | 0.1 (0-0.1) | 0.2 (0.1-0.3) | 4905.9 (1988.6-8172.9) | 34901.3 (14486.2-57402.6) | 2 (0.8-3.3) | 5.2 (2.2-8.6) | 3.28 (3.2-3.36) | 3.27 (3.23-3.31) |
| Low SDI | 24.3 (7.9-49.9) | 161.3 (63.8-308.1) | 0 (0-0.1) | 0.1 (0-0.2) | 976.8 (346.2-1753.7) | 5881.3 (2413.3-10532.8) | 1 (0.3-1.8) | 2.6 (1.1-4.7) | 3.37 (3.14-3.6) | 3.17 (3.03-3.3) |
| Region | | | | | | | | | | |
| Central Europe | 401.1 (178.7-672.1) | 1002.4 (452.7-1727.3) | 0.5 (0.2-0.9) | 0.6 (0.3-1.1) | 10069.8 (4074.4-17323.5) | 21849.7 (9300.9-38053.6) | 12 (4.8-20.4) | 14.8 (6.3-25.8) | 0.69 (0.45-0.93) | 0.63 (0.47-0.79) |
| Western Europe | 1499.2 (624.9-2563.5) | 4799.7 (1972.2-8610.8) | 0.4 (0.2-0.6) | 0.6 (0.2-1) | 33293.2 (12973.8-55762.6) | 83359 (33784.9-145732.1) | 8.6 (3.4-14.5) | 12.4 (5.1-21.8) | 1.57 (1.48-1.67) | 1.22 (1.11-1.33) |
| Eastern Europe | 654.6 (283-1088.7) | 1767.7 (754.6-3046.4) | 0.4 (0.2-0.7) | 0.7 (0.3-1.2) | 18737.5 (7666.4-32170.5) | 41637.5 (17830.1-70933.4) | 10.3 (4.2-17.6) | 17.3 (7.4-29.6) | 1.65 (1.48-1.82) | 1.63 (1.5-1.77) |
| Central Asia | 50.9 (21.6-85.7) | 122.2 (54.6-205.3) | 0.2 (0.1-0.3) | 0.3 (0.1-0.5) | 1842.9 (748.9-3192.6) | 4100.1 (1706.5-7233.4) | 6.9 (2.8-11.9) | 9.9 (4.1-17.2) | 1.27 (1.04-1.51) | 1.08 (0.99-1.16) |
| East Asia | 44.7 (10.2-98.9) | 1908.4 (745.2-3436.3) | 0 (0-0) | 0.2 (0.1-0.3) | 1604.7 (438.9-3313.2) | 52207.1 (20096.4-92152.4) | 0.4 (0.1-0.9) | 4.7 (1.8-8.2) | 7.84 (7.6-8.07) | 7.99 (7.84-8.15) |
| South Asia | 19.8 (5.3-48) | 522 (207.4-1025.3) | 0 (0-0) | 0.1 (0-0.2) | 1024.1 (340.9-1986.2 | 18908.3 (7805.7-34250.8) | 0.4 (0.1-0.8) | 2.7 (1.1-5) | 7.62 (7.41-7.83) | 6.69 (6.5-6.88) |
| Southeast Asia | 18.2 (3.8-38.4) | 414.1 (157.4-741) | 0 (0-0) | 0.2 (0.1-0.3) | 973.1 (319.3-1828.3) | 14161.7 (5544.3-23710.6) | 0.7 (0.2-1.4) | 4.3 (1.7-7.3) | 7.3 (7.05-7.55) | 6.05 (5.84-6.25) |
| High-income Asia Pacific | 22.5 (7.3-47.6) | 235.2 (81.5-455.5) | 0 (0-0) | 0.1 (0-0.1) | 777.1 (246-1609.9) | 4991.1 (1863.7-9059.1) | 0.7 (0.2-1.4) | 1.7 (0.6-3) | 2.61 (2.25-2.97) | 2.79 (2.32-3.26) |
| High-income North America | 842.8 (333.8-1590.9) | 3223.9 (1326.2-5541.2) | 0.3 (0.1-0.6) | 0.7 (0.3-1.2) | 24090 (9538.4-44059) | 77878.5 (33047.3-132858.3) | 10.8 (4.3-19.7) | 19.2 (8.2-32.4) | 2.02 (1.85-2.19) | 1.68 (1.53-1.83) |
| Caribbean | 26.8 (11.8-44.6) | 144.6 (63.3-242.3) | 0.2 (0.1-0.4) | 0.4 (0.2-0.8) | 824.4 (341.1-1389) | 3563.1 (1462.1-6217.2) | 6.5 (2.7-11) | 11.9 (4.9-20.8) | 1.91 (1.81-2.01) | 1.92 (1.85-1.98) |
| Tropical Latin America | 83.7 (31.4-157) | 804.8 (329.7-1396.3) | 0.2 (0.1-0.5) | 0.6 (0.2-1) | 3271.4 (1264.7-5855.4) | 20417 (8363.6-35447.7) | 7.4 (2.8-13.3) | 14.3 (5.8-24.8) | 2.67 (2.41-2.92) | 2 (1.88-2.12) |
| Central Latin America | 108.2 (45.4-196.6) | 777.8 (339.3-1359.5) | 0.3 (0.1-0.6) | 0.6 (0.3-1) | 3611.8 (1440.1-6525.3) | 20665.8 (8938.6-36725) | 9.4 (3.7-17.1) | 15.6 (6.7-27.6) | 1.86 (1.79-1.94) | 1.63 (1.6-1.66) |
| Andean Latin America | 11.3 (4.1-22.7) | 110.5 (45-200.4) | 0.1 (0-0.3) | 0.4 (0.1-0.7) | 453.9 (165.5-877.5) | 3290.1 (1321.8-5794) | 4.4 (1.6-8.6) | 10.8 (4.3-18.9) | 3.48 (3.31-3.66) | 2.88 (2.81-2.96) |
| Southern Latin America | 68.7 (28.4-130.8) | 279.8 (111.5-512.7) | 0.3 (0.1-0.6) | 0.5 (0.2-0.9) | 1799 (708.4-3252.5) | 5319.9 (2107.2-9666.5) | 7.2 (2.8-13) | 9.8 (3.9-17.7) | 2.39 (1.95-2.82) | 1.51 (1.24-1.77) |
| Oceania | 2 (0.8-4) | 8.5 (3.7-15.3) | 0.2 (0.1-0.5) | 0.3 (0.2-0.6) | 84.2 (34.2-154.7) | 329.3 (140.4-573) | 6.2 (2.4-12.2) | 9.8 (4.2-17.2) | 1.34 (1.26-1.42) | 1.41 (1.35-1.47) |
| Australasia | 68.3 (27.5-131.8) | 349 (144.4-618.3) | 0.5 (0.2-1) | 0.9 (0.4-1.6) | 1525.1 (617-2814) | 6426.8 (2722-11079.5) | 11.2 (4.6-20.6) | 19.2 (8.1-32.8) | 1.99 (1.76-2.23) | 1.77 (1.64-1.91) |
| Central Sub-Saharan Africa | 3.7 (1.3-7.7) | 40.9 (15.1-86.8) | 0 (0-0.1) | 0.2 (0.1-0.5) | 139.6 (52.8-264.6) | 1292.9 (495.6-2450) | 1.3 (0.5-2.5) | 5.2 (1.9-10.1) | 4.85 (4.7-5) | 4.46 (4.38-4.53) |
| Eastern Sub-Saharan Africa | 6.6 (1.9-14) | 59.1 (22.2-116.8) | 0 (0-0.1) | 0.1 (0-0.2) | 319 (105.6-620.2) | 2511 (1029.6-4471.8) | 0.9 (0.3-1.8) | 3.1 (1.3-5.6) | 4.56 (4.39-4.72) | 4.13 (4.04-4.22) |
| North Africa and Middle East | 193.6 (78.7-347.7) | 1144.7 (480-1924.5) | 0.4 (0.1-0.7) | 0.8 (0.3-1.3) | 5072.8 (2019.9-9000.3) | 25807.3 (11358.6-43877.1) | 7.5 (3-13.5) | 14.4 (6.2-24.4) | 2.79 (2.45-3.13) | 2.14 (1.93-2.34) |
| Southern Sub-Saharan Africa | 28.7 (12.3-50.2) | 156.6 (72.8-264.9) | 0.2 (0.1-0.4) | 0.6 (0.3-1.1) | 1110.5 (463.1-1881.5) | 4643 (2071.8-7961.2) | 7.9 (3.2-13.3) | 15.8 (7.1-26.7) | 3.24 (2.84-3.63) | 2.32 (2.13-2.51) |
| Western Sub-Saharan Africa | 38.2 (14.8-73.5) | 186.7 (76.5-324.6) | 0.1 (0-0.3) | 0.3 (0.1-0.6) | 1027.6 (422.6-1800.9) | 5276.3 (2157.2-9081.8) | 2.9 (1.1-5.2) | 6.8 (2.8-11.5) | 2.38 (2.18-2.59) | 2.46 (2.33-2.6) |
| Abbreviations: AF: atrial fibrillation; high BMI: high body mass index; EAPC: estimated annual percentage change; ASDR: age standardized death rate; DALYs: disability-adjusted life years; ASRDLAYs: age-standardized rate of disability-adjusted life years; SDI: sociodemographic index; UI: uncertainty interval; CI: confidence interval. | | | | | | | | | | |

| **Table S3.** Deaths, ASDR, ASRDALYs of AF burden attributable to high BMI of each age group (30-95+ years, 5-year intervals) in 1990 and 2021. | | | | | | | | |
| --- | --- | --- | --- | --- | --- | --- | --- | --- |
|  | Deaths (95% UI) | | ASDR (95% UI) | | DALYs (95% UI) | | ASRDALYs (95% UI) | |
|  | 1990 | 2021 | 1990 | 2021 | 1990 | 2021 | 1990 | 2021 |
| 30-34 | 2.1 (0.9-3.5) | 8.2 (3.6-13.3) | 0 (0-0) | 0 (0-0) | 191.6 (76.7-338) | 728 (301.3-1215.7) | 0 (0-0.1) | 0.1 (0-0.2) |
| 35-39 | 3.7 (1.5-6.3) | 14.7 (6.6-23.6) | 0 (0-0) | 0 (0-0) | 708.6 (269.9-1376.5) | 2591.7 (1059.8-4885.1) | 0.2 (0.1-0.4) | 0.5 (0.2-0.9) |
| 40-44 | 11 (4.5-19) | 44.3 (20.3-71.3) | 0 (0-0) | 0 (0-0) | 1918.7 (720.3-3758.7) | 7089.3 (2893.2-12970.2) | 0.7 (0.3-1.3) | 1.4 (0.6-2.6) |
| 45-49 | 26.3 (10.7-46.3) | 107.5 (48.9-174.9) | 0 (0-0) | 0 (0-0) | 3663.7 (1437.3-7037) | 14864.5 (6227-27008.2) | 1.6 (0.6-3) | 3.1 (1.3-5.7) |
| 50-54 | 62.2 (25.7-110.4) | 241.8 (108.9-395.8) | 0 (0-0.1) | 0.1 (0-0.1) | 7045.6 (2724-13435.2) | 27716.4 (11425.2-51574) | 3.3 (1.3-6.3) | 6.2 (2.6-11.6) |
| 55-60 | 122.6 (50.9-211.8) | 484.3 (219.7-786.7) | 0.1 (0-0.1) | 0.1 (0.1-0.2) | 11269.2 (4752.8-20929.8) | 45494.3 (20198.2-82578.4) | 6.1 (2.6-11.3) | 11.5 (5.1-20.9) |
| 61-64 | 234.6 (99.9-409.8) | 814 (365-1321.8) | 0.1 (0.1-0.3) | 0.3 (0.1-0.4) | 17937.7 (7025.9-32398.7) | 64434.9 (27736.2-112479.7) | 11.2 (4.4-20.2) | 20.1 (8.7-35.1) |
| 65-69 | 351 (149.2-633.9) | 1275 (575.6-2082.8) | 0.3 (0.1-0.5) | 0.5 (0.2-0.8) | 23147.2 (8887.5-40917.7) | 86718.8 (35838.3-152237.7) | 18.7 (7.2-33.1) | 31.4 (13-55.2) |
| 70-74 | 518 (214.3-911.8) | 2219.2 (983.4-3693.3) | 0.6 (0.3-1.1) | 1.1 (0.5-1.8) | 24797.6 (9402.7-43119.1) | 106639 (43202-179214.6 | 29.3 (11.1-50.9) | 51.8 (21-87.1) |
| 75-79 | 917.2 (382-1621.7) | 2989.8 (1323.6-4936.6) | 1.5 (0.6-2.6) | 2.3 (1-3.7) | 30475.2 (11779.6-53483) | 100515.9 (41321.4-171464.6) | 49.5 (19.1-86.9) | 76.2 (31.3-130) |
| 80-84 | 1182.3 (487.7-1987.2) | 4712.2 (2016.1-8090.7) | 3.3 (1.4-5.6) | 5.4 (2.3-9.2) | 24943.6 (10037.7-41590.7) | 98237.4 (39936.4-167241.5) | 70.5 (28.4-117.6) | 112.2 (45.6-191) |
| 85-89 | 1166.1 (469.2-1970.2) | 5816.7 (2455.2-10147.6) | 7.7 (3.1-13) | 12.7 (5.4-22.2) | 17082.7 (6657.5-28875.4) | 82860.8 (33750.5-141573.9) | 113 (44.1-191.1) | 181.2 (73.8-309.6) |
| 90-94 | 820.4 (331.7-1396.2) | 5773.1 (2423.6-10126.8) | 19.1 (7.7-32.6) | 32.3 (13.5-56.6) | 8906.8 (3499.4-15164.2) | 61111.4 (25180.7-105602.7) | 207.9 (81.7-353.9) | 341.6 (140.8-590.3) |
| 95+ | 304.2 (124.3-530.7) | 2735.9 (1120.2-4773.8) | 29.9 (12.2-52.1) | 50.2 (20.6-87.6) | 2943.5 (1172.1-5140) | 25571.1 (10631.8-44227.7) | 289.1 (115.1-504.9) | 469.2 (195.1-811.5) |
| Abbreviations: AF: atrial fibrillation; high BMI: high body mass index; ASDR: age standardized death rate; DALYs: disability-adjusted life years; ASRDLAYs: age-standardized rate of disability-adjusted life years; UI: uncertainty interval; | | | | | | | | |

| **Table S4**. The death number of three age groups in different regions from 1990-2021. | | | | | | |
| --- | --- | --- | --- | --- | --- | --- |
|  | 30-49 years | | 50-69 years | | 70-89 years | |
|  | 1990(95% UI) | 2021(95% UI) | 1990(95% UI) | 2021(95% UI) | 1990(95% UI) | 2021(95% UI) |
| Global | 43.1 (17.6-75) | 174.7 (79.5-283.2) | 770.5 (325.7-1365.9) | 2815.2 (1269.2-4587.1) | 4604 (1884.9-7887.1) | 21511 (9201.9-36994.9) |
| SDI | | | | | | |
| High SDI | 16.4 (6.9-29.6) | 46.4 (21.3-75.4) | 364 (150.4-668.8) | 1036.4 (471-1706.3) | 2743 (1131-4850.4) | 10282.5 (4340.8-18231.8) |
| High-middle SDI | 8.3 (3.3-14.5) | 25.5 (11-44.5) | 254.4 (106.6-434.1) | 690.1 (303.2-1180.6) | 1378.7 (572.8-2325.1) | 5869.9 (2483.9-10077.6) |
| Middle SDI | 12.5 (5.2-21.1) | 63.5 (28.7-104.7) | 96 (39.9-166) | 693.3 (310.7-1157.9) | 312 (118.9-546) | 3826.3 (1655.7-6438.7) |
| Low-middle SDI | 4.8 (2-8.4) | 31.4 (14-51) | 42.5 (18-73.5) | 319.7 (141.3-517.2) | 143 (55.1-251.7) | 1322.8 (560.6-2212.9) |
| Low SDI | 0.9 (0.3-1.9) | 7.6 (3-13.6) | 11.5 (3.8-23.1) | 71.8 (27.7-131.2) | 17.4 (4.6-37.4) | 178.7 (68-332.8) |
| Region | | | | | | |
| Central Europe | 2.4 (1-4.3) | 2.7 (1.1-4.8) | 98.9 (42-174.9) | 143.9 (62.3-249.6) | 456.4 (196.8-802.3) | 1222.8 (536.1-2180.6) |
| Western Europe | 6.3 (2.5-11.6) | 9.8 (4-17.7) | 170.6 (67.5-323.6) | 311.2 (127.9-572.7) | 1762.8 (738.4-3036.8) | 5866 (2397.4-10585.4) |
| Eastern Europe | 2.2 (0.9-3.8) | 6.1 (2.5-10.6) | 120.5 (51.1-201.7) | 272.3 (116.8-462) | 636.7 (267.7-1082.7) | 1882.9 (815-3237.8) |
| Central Asia | 0.5 (0.2-0.9) | 1.6 (0.7-3) | 12.3 (5.3-22.1) | 36.3 (15.2-64.2) | 44.5 (18.4-77.3) | 117.8 (51.5-200.3) |
| East Asia | 0.9 (0.2-1.9) | 12.4 (5-24.5) | 15.9 (4.5-33.7) | 308.1 (125.3-574.3) | 40.3 (5.2-97.5) | 2032 (808.6-3639.8) |
| South Asia | 1.5 (0.5-3.3) | 16.4 (6.7-28.6) | 10.5 (2.7-24) | 188.6 (72.8-343.7) | 18.1 (3-46.9) | 591.5 (216.3-1194.7) |
| Southeast Asia | 2 (0.6-4) | 15.3 (6.3-25.8) | 8.6 (2.6-16.8) | 100.7 (42.7-170.4) | 10.8 (-0.4-28.8) | 360.8 (122.2-687.5) |
| High-income Asia Pacific | 0.5 (0.1-1) | 1.5 (0.6-2.8) | 6.7 (2-13.7) | 26.1 (10.2-49.9) | 23.5 (5.7-54.6) | 272.8 (91-542.5) |
| High-income North America | 8.7 (3.6-15.5) | 28.4 (13.2-45.5) | 160 (65.9-292.7) | 619.2 (281.6-983) | 958.3 (384-1866.6) | 4313.8 (1781.9-7592.4) |
| Caribbean | 0.8 (0.4-1.4) | 2.5 (1.1-4.3) | 6.7 (2.8-11.5) | 26 (11.4-44.4) | 29.2 (11.7-49) | 167.8 (72.9-282.1) |
| Tropical Latin America | 3.2 (1.2-5.9) | 15.1 (6.4-25.8) | 25.5 (10.1-48.6) | 148.1 (62.4-260.2) | 83 (28.4-163.4) | 990.4 (401.3-1760.6) |
| Central Latin America | 3.6 (1.5-6.7) | 16.1 (7.1-27.7) | 29.7 (12.1-54.3) | 162.9 (70-282) | 117.5 (46.5-218) | 969.5 (417.1-1739) |
| Andean Latin America | 0.8 (0.3-1.6) | 2.9 (1.3-5.3) | 5.7 (2.1-11.5) | 27 (11.8-48.4) | 13 (4.2-27.2) | 139.6 (56.2-256.7) |
| Southern Latin  America | 0.9 (0.3-1.7) | 2.1 (0.9-3.6) | 13.5 (5.3-24.6) | 36.8 (15.2-62.4) | 77.3 (30.4-146.6) | 338.2 (138.5-622.7) |
| Oceania | 0.4 (0.2-0.8) | 1.6 (0.7-3.1) | 1.4 (0.6-2.7) | 5.6 (2.3-9.9) | 1.2 (0.4-2.4) | 6.3 (2.6-11.2) |
| Australasia | 0.5 (0.2-0.9) | 1.4 (0.6-2.4) | 10.3 (4.1-19.2) | 32.7 (14-54.6) | 80.1 (31.1-151.6) | 454.8 (187.7-833.4) |
| Central Sub-Saharan Africa | 0.1 (0-0.3) | 1.8 (0.6-3.6) | 2.7 (0.9-5.9) | 23.9 (8.4-49.3) | 2.3 (0.4-5.8) | 40.8 (13.3-85.5) |
| Eastern Sub-Saharan Africa | 0.3 (0.1-0.7) | 3.1 (1.1-5.9) | 3.4 (1-7.6) | 28.3 (10.2-54.4) | 4.5 (0.8-11.1) | 57.8 (20.2-111.7) |
| North Africa and Middle East | 5.4 (2.2-9.8) | 26 (11.7-43.6) | 53 (22.5-94.8) | 241.3 (107.8-399.4) | 179.1 (69.6-320.4) | 1285.3 (541.1-2211) |
| Southern Sub-Saharan Africa | 1.7 (0.7-2.9) | 5.9 (2.5-10) | 7 (3.1-11.9) | 39 (17.4-65) | 25.8 (10.5-46.7) | 155.8 (69.8-261.6) |
| Western Sub-Saharan Africa | 0.4 (0.1-0.6) | 2.1 (0.8-3.7) | 7.7 (3.1-13.9) | 37 (14.4-64.7) | 39.7 (13.4-79.1) | 244.3 (102.8-425.5) |
| Abbreviations: AF: atrial fibrillation; high BMI: high body mass index; ASDR: age standardized death rate; DALYs: disability-adjusted life years; ASRDLAYs: age-standardized rate of disability-adjusted life years; SDI: sociodemographic index; UI: uncertainty interval. | | | | | | |

**Fig.S1** Global burden of high BMI-related AF among 204 countries and territories by sex in 2021, related to Fig.1. (A-D) death number, ASDR, DALYs and ASRDALYs in female; (E-H) death number, ASDR, DALYs and ASRDALYs in male.

**Fig.S2** Temporal trends of AF burden attributable to high BMI by gender from 1990 to 2021, related to Fig.2. (A-B) Death number and DALYs of AF burden attributable to high BMI; (C-D) ASDR and ASRDALYs of AF burden attributable to high BMI.

**Fig.S3** Sex differences and trends in high BMI-related AF in different regions from 1990 to 2021, related to Fig.4. (A) The ASRDALYs trends with low to high SDIs in male and female, respectively; (B) Male to female ratios of ASRDALYs in five SDI regions. (C-D) EAPC of ASRDALYs in 204 countries and territories in female and male.

**Fig.S4** AF burden attributable to high BMI and the trends by age groups in different regions, related to Fig.5. (A) The ASRDALYs changes in each age group (30–95+ years, 5-year intervals) in SDI quintiles; (B-C) The trends of DALYs in each age group from 1990 to 2021 with high to low SDI regions; (D) The three age groups as proportions of total DALYs globally.
